# Comparative miRNAs analysis of Two contrasting broccoli inbred lines with divergent head-forming capacity under temperature stress

**DOI:** 10.1186/s12864-015-2201-1

**Published:** 2015-12-01

**Authors:** Chi-Chien Chen, Shih-Feng Fu, Monma Norikazu, Yau-Wen Yang, Yu-Ju Liu, Kazuho Ikeo, Takashi Gojobori, Hao-Jen Huang

**Affiliations:** Department of Life Sciences, National Cheng Kung University, No. 1 University Road, East Dist, Tainan, 701 Taiwan; Department of Biology, National Chunghua University of Education, No.1, Jin-De Road, Changhua, 500 Taiwan; Center for Information Biology and DNA Data Bank of Japan, National Institute of Genetics, Yata, Mishima, Shizuoka, 411-8540 Japan; Kale Biotech. Co, No.68-1, Chongde 16th St., East Dist, Tainan, 701 Taiwan

**Keywords:** Broccoli, Deep sequencing, Genotype, Head-forming capacity, High temperature, MicroRNA

## Abstract

**Background:**

MicroRNAs (miRNAs) play a vital role in growth, development, and stress response at the post-transcriptional level. Broccoli (*Brassica oleracea* L. var *italic*) is an important vegetable crop, and the yield and quality of broccoli are decreased by heat stress. The broccoli inbred lines that are capable of producing head at high temperature in summer are unique varieties in Taiwan. However, knowledge of miRNAomes during the broccoli head formation under heat stress is limited.

**Methods:**

In this study, molecular characterization of two nearly isogenic lines with contrasting head-forming capacity was investigated. Head-forming capacity was better for heat-tolerant (HT) than heat-sensitive (HS) broccoli under heat stress.

**Results:**

By deep sequencing and computational analysis, 20 known miRNAs showed significant differential expression between HT and HS genotypes. According to the criteria for annotation of new miRNAs, 24 novel miRNA sequences with differential expression between the two genotypes were identified. To gain insight into functional significance, 213 unique potential targets of these 44 differentially expressed miRNAs were predicted. These targets were implicated in shoot apical development, phase change, response to temperature stimulus, hormone and energy metabolism. The head-forming capacity of the unique HT line was related to autonomous regulation of *Bo-FT* genes and less expression level of heat shock protein genes as compared to HS. For the genotypic comparison, a set of miRNAs and their targets had consistent expression patterns in various HT genotypes.

**Conclusions:**

This large-scale characterization of broccoli miRNAs and their potential targets is to unravel the regulatory roles of miRNAs underlying heat-tolerant head-forming capacity.

**Electronic supplementary material:**

The online version of this article (doi:10.1186/s12864-015-2201-1) contains supplementary material, which is available to authorized users.

## Background

During recent decades, global warming and climate change have been important topics in modern biology. The world’s average temperature, atmospheric CO_2_ concentration, and tropospheric ozone concentration ([O_3_]) are increasing, thus leading to climatic extremes [1]. High temperature exposure results in reduced plant growth and productivity, including broccoli, tomato and wheat [2–5]. Broccoli, *Brassica oleracea* L. var *italica*, is an important vegetable crop containing multiple nutrients and anti-cancer phytochemicals such as glucoraphanin and its derivative sulforaphane [6]. Floral heads of broccoli are harvested at an average daily temperature below 22 °C. Broccoli head is characterized by a fused inflorescence of several arrested floral spikes with proliferation of almost fully developed floral buds. The head formation of broccoli is sensitive to and irreversible with high temperature, so the yield and quality of the crop decreases [7, 8]. Heat stress is limiting for the production of vegetable crops in many areas of the world. Therefore, the investigation of molecular mechanisms into heat-tolerant head-forming capacity in broccoli can help decipher the biological basis of the response to heat and the selection of broccoli with improved heat tolerance.

The transition from the vegetative to reproductive phase during plant development is strictly intermingled in response to environmental and endogenous cues [9, 10]. Temperature and photoperiod are the two major environmental cues that influence i) duration of juvenile period (pre-vernalization), ii) those for floral initiation, iii) for development of floral primordia, iv) inflorescence organ identity and v) final floral development (i.e. beyond the typical transitory arrest of a commercial broccoli). The acceleration of flowering by prolonged exposure to low temperature is called vernalization. Without environmental signals, endogenous cues include the developmental or autonomous pathway that still can modulate flowering time [10]. Leaf primordia derive from the peripheral zone of the shoot apical meristem (SAM) during the vegetative phase. Then, cauline leaf primordia subtend axillary inflorescence meristems in the early reproductive phase and the floral primordia form a bractless flower during the late reproductive phase [10]. Molecular genetic analyses have revealed that *FLOWERING LOCUS C* (*FLC)* is a major flowering repressor in the autonomous and vernalization pathways regulating flowering time in *Arabidopsis*. Expressing *FLC* from the phloem or SAM strongly inhibits both *FLOWERING LOCUS T* (*FT*) and *SUPPRESSOR OF OVEREXPRESSION OF CONSTANS1* (*SOC1*) expression by directly binding to their regulatory regions. *FT* and *SOC1* activate the specification of floral organ identity genes, such as *CAULIFLOWER* (*CAL*) and *APETALA1* (*AP1*) that are required for flower formation in the meristem [9]. Despite a highly conserved molecular mechanism for the precise orchestration of flowering time, species-specific differences exist in signal perception, transduction and integration in flowering time pathways [11]. Further open questions are how these networks evolved and the topology of the gene networks in different species.

Endogenous small non-coding RNAs are of four groups -- microRNAs (miRNAs), trans-acting small interfering RNAs, natural antisense transcripts siRNAs, and repeat-associated siRNAs -- and are implicated in the plant response to abiotic stress [12]. Plant miRNAs are 20–to 24–nt single-stranded RNAs and are processed from stem-loop precursors by the ribonuclease III Dicer-like I (DCL1) and its interacting partner, the RNA binding protein HYPONASTIC LEAVES1 (HYL1) [13]. miRNAs negatively regulate the gene expression of target mRNAs at the nearly perfect base complementarity via endonucleolytic cleavage or translational inhibition at the post-transcriptional level [13]. Evidence indicates that miRNAs have various functions during plant development in response to environmental signals and are essential and effective regulators [14–18]. In one of the pioneering studies, miR156 was found to target *SQUAMOSA PROMOTER BINDING PROTEIN LIKE 9* (*SPL9*), and miR172b was found to target *APETALA2-LIKE* (*AP2-like*) transcription factors (*TOE1* and *TOE2*) to regulate the temporal coordination of vegetative phase change and floral induction [15, 16]. Genome-wide analysis of miRNAs showed that miR156, miR159, miR160, miR166, miR168, miR169, miR827, and miR2005 are induced by heat stress in wheat [17]. Many miRNA-target genes were discovered, and the identification of lower-level non-conserved miRNAs was feasible by high-throughput degradome sequencing and bioinformatics analysis in various species [17–19].

In all, 863 miRNAs have been identified in *Brassicaceae*, including *Arabidopsis thaliana*, *Brassica napus* and *Brassica rapa*, and are deposited in the miRNA database miRBase (Release 21, Jun. 2014; http://www.mirbase.org/index.shtml) [20]. One of these miRNAs, miR156, induced at early embryonic developmental stages in seeds, could target *SPL* family genes in Arabidopsis [16]*.* Overall, 21 novel miRNAs were identified by deep sequencing (Solexa) technology; bra-miR5714 and bra-miR5726 were found to be up-regulated and bra-miR5716 and bra-miR1885b.3 down-regulated under heat stress in *B. rapa*. The predicted target of bra-miR1885b.3 is involved in the regulation of plant heat tolerance [18]. A total of 168 potential miRNAs belonging to 38 miRNA families were predicted by computational analysis of *Brassica rapa* subsp. *pekinensis* [21]. Many predicted miRNA targets encode transcription factors that play vital roles in plant development. For example, the miR1533 family is the largest family of miRNAs and has 52 potential target genes including DRE-binding transcription factor, NAC-domain protein, and WRKY transcription factors [21]. Although hundreds of plant miRNAs have been identified, only 19 conserved miRNA families were reported in *B. oleracea* by experimental or computational studies [14, 22]. Previous studies have demonstrated the importance of temperature requirements for flowering in *Brassica* plant species [3, 7]. However, the regulatory roles of broccoli miRNAs with changed expression profiles during head formation in response to heat stress have yet to be thoroughly explored.

In this study, the broccoli inbred lines that are capable of undergoing floral initiation to produce head at high temperature are unique varieties in Taiwan. The head-forming capacity at high temperature of the desired traits is developed through natural selection followed by conventional breeding programs [5]. However, the molecular mechanisms underlying the head-forming capacity at high temperature in broccoli remain to be elusive. It is important to identify novel miRNAs and uncovered differently expressed miRNAs in two representative broccoli genotypes to deduce the molecular regulation underlying head-forming capacity at high temperature. Two comprehensive small RNA (sNRA) libraries from the two broccoli genotypes were constructed by a deep sequencing approach. One broccoli genotype exhibited heat-tolerant head-forming capacity (HT) and the other line is heat-sensitive (HS). Overall, 53,322,939 total sequence reads representing 19,202,332 unique sRNAs were sequenced, and most were 23 to 24 nt in length. From sequencing library information and bioinformatics analysis, 20 known and 24 novel miRNAs with significantly differential expression between the HT and HS genotypes were discovered. The potential targets of the differentially expressed miRNAs were predominantly involved in development, response to oxidative stress, and energy metabolism. Here the regulatory interactions between miRNAs and their target genes provide new insights into miRNAs in broccoli associated with heat-tolerant head-forming capacity.

## Results

### Head-forming capacity changes under different temperature in two broccoli genotypes

**Fig. 1:**
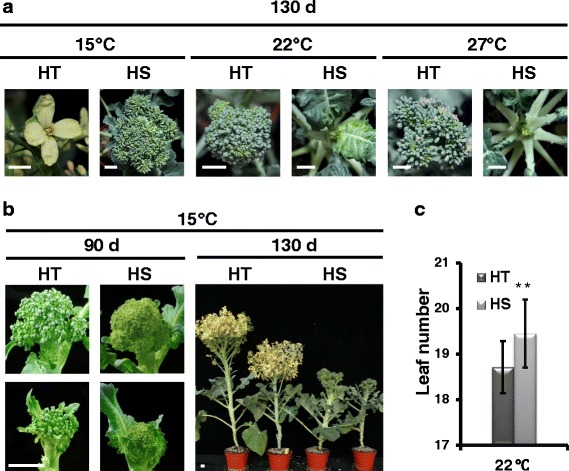
Differential heat tolerance of two broccoli lines. **a** Head-forming capacity of heat-tolerant 'B295' (HT) and heat-sensitive 'BR1 op' (HS) broccoli (*B. oleracea* L. var *italica*) under different temperatures (15, 22 and 27 °C) at 130 days post-germination (DPG). Scale bars = 1 cm. **b** Head-forming capacity of HT and HS genotypes under 15 °C at 90 and 130 DPG, respectively. **c** Total leaf number under long-day conditions at 22 °C (Student’s *t*-test, **P < 0.01)

To understand the relationship between head-forming capacity and temperature in two HT and HS broccoli genotypes, the genotypes were grown under different temperatures under long-day conditions. The temperature treatment was applied to plants at 40 days post-germination (DPG) (Fig. 1a). HT and HS genotypes showed similar head-forming capacity at 15 °C at 90 DPG (Fig. 1b). The head of HT genotype continued to produce completely opened flower as compared with clusters of tight green flower buds of HS at 130 DPG (Fig. 1b). Both HT and HS genotypes exhibited head-forming capacity at 15 °C, but only the HT genotype showed head-forming capacity at 22 °C and 27 °C at 130 DPG (Fig. 1a). Leaf numbers at head formation were lower in the HT than HS line at 22 °C at 130 DPG (Fig. 1c). The HS line failed to produce heads at 22 °C throughout cultivation.

### Overview of sRNA sequencing

To examine sRNAs linked to the regulation of head-forming capacity in the two genotypes, deep sequencing were used to construct two sRNA libraries from shoot apexes of the HT and HS genotypes at 50 DPG at 22 °C prior to head formation. The total sequence reads of 26.7 million (range 26.2–27.1 million), on average, from each library were obtained after discarding the adapter and low-quality sequences (Table 1). The mean number of unique reads in the two libraries was 9.6 million (range: 9.5–9.7 million). Sequences were aligned to the collected *Brassica*-related transcriptomic and genomic libraries. A total of 86.2 and 86.1 % sRNA sequences, representing 79.6 and 79.6 % of unique sequences, respectively, were perfectly mapped for further computational analysis. About 0.2 and 0.2 %, respectively, of the unique sequences as known miRNAs were identified, which account for 4.1 and 3.3 %, respectively, of the total sRNAs. sRNA reads that might be from known rRNA, tRNA, small nuclear RNA (snRNA), and small nucleolar RNA (snoRNA) were excluded. The remaining unique sequences (77.2 and 77.3 %, respectively) could be used to identify novel miRNA candidates.

**Table 1:** Different categories of small RNAs from HT and HS broccoli under heat stress

Category	HT		HS	
	Total	Unique	Total	Unique
All read	27,069,903 (100.0 %)	9,459,174 (100.0 %)	26,253,036 (100.0 %)	9,743,157 (100.00 %)
Matched to *Brassica rapa* genome	19,365,028 (71.5 %)	6,090,652 (64.4 %)	18,657,531 (71.1 %)	6,273,570 (64.4 %)
Matched to *Brassica* EST	23,322,757 (86.2 %)	7,533,369 (79.6 %)	22,610,232 (86.1 %)	7,759,418 (79.6 %)
Non-coding RNAs (rRNA, tRNA, etc.)	3,325,500 (12.3 %)	225,054 (2.4 %)	2,591,009 (9.9 %)	219,386 (2.3 %)
Known miRNAs	1,098,316 (4.1 %)	16,065 (0.2 %)	854,848 (3.3 %)	16,527 (0.2 %)
Candidates for novel miRNA prediction	19,973,881 (73.8 %)	7,303,071 (77.2 %)	19,983,768 (76.1 %)	7,534,627 (77.3 %)
Unannotated RNAs	2,675,273 (9.9 %)	1,379,348 (14.6 %)	2,603,298 (9.9 %)	1,420,384 (14.6 %)

*EST* expressed sequence tag, *HT* heat-tolerant broccoli, *HS* heat-sensitive broccoli

**Fig. 2:**
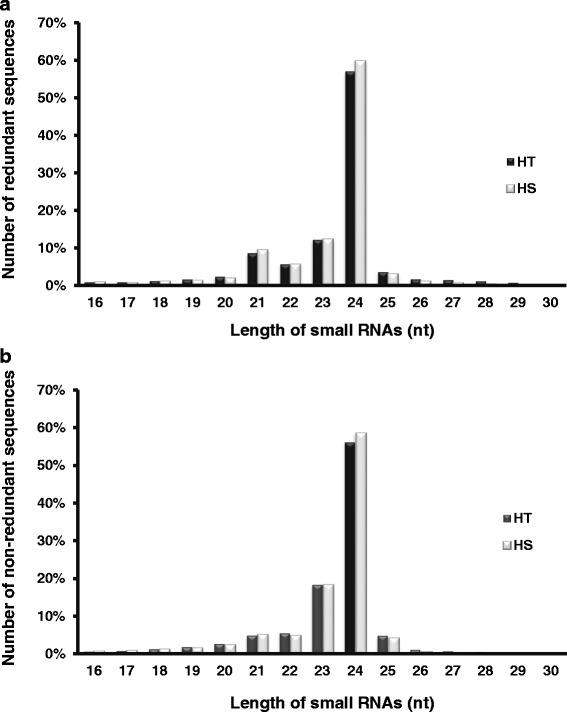
Length distribution of redundant (**a**) and non-redundant (**b**) small RNA sequences. Data from shoot apexes of HT and HS broccoli. HT: heat-tolerant broccoli, HS: Heat-sensitive broccoli, nt, nucleotides

sRNAs showed wide variation in length, from 16 to 30 nt (Fig. 2), with three major peaks at 21–, 23–, and 24-nt in the redundant sequence reads (Fig. 2a). In the two libraries, the major classes of total sRNAs were 24 nt: 57.1 and 59.8 %, respectively. The 24-nt sRNAs were also predominant in the non-redundant sequences, implying that the sRNAs sequences are diverse in broccoli (Fig. 2b).

### Known miRNAs in broccoli

Use of computational algorithms revealed 161 known miRNAs belong to 51 families with an average of about three miRNA members per family (Additional file 1). The size distribution of known miRNAs were mostly 21 nt (95 of 161, 59.1 %). A total of 51 corresponding miRNA* sequences for known miRNAs were detected (Additional file 1). Although most were present at a very low expression, the abundance was higher for some miRNAs* than their corresponding miRNAs. In particular, the expression of miR398b* was greater by 5.18-fold that of *miR398b* in the HT library in transcripts per million (TPM). An advantage of deep sequencing is the ability to determine individual members within a miRNA family. In general, the Solexa sequencing results showed similar expression of members in the same family, such as *miR160* and *miR171* families. The sequencing analysis also revealed varied expression of some members in certain families (Additional file 1). In the HT library, the expression of the miR319 family ranged from 0.15 TPM (*miR319b*) to 307.1 TPM (*miR319g*) and that of the *miR169* family from 0.04 TPM (*miR169d*) to 40.93 TPM (*miR169m*). In addition, *miR164a* was more highly expressed than other members of the *miR164* family in both the HT and HS libraries.

### Expression profiling of known miRNAs

To determine which miRNAs correlated with the heat-tolerant head-forming capacity, the expression of miRNAs in the HT and HS lines were compared. There were 20 differentially expressed known miRNAs in the HT and HS libraries (Table 2). In the HT genotype, the expression of *miR156/miR157*, *miR169*, *miR172*, *miR395*, and *miR408* were significantly up-regulated, whereas that of *miR166*, *miR391*, *miR398*, *miR400*, and *miR827* was down-regulated as compared with the HS genotype. Most of the miRNAs showed greater expression in the HT than HS genotype. The differential expression profiles of these miRNAs in the two genotypes may contribute to heat-tolerant head-forming capacity.

**Table 2:** Differentially expressed known miRNAs in shoot apexes between HT and HS broccoli under heat stress

Family	miRNA	miRNA sequence (5′- > 3′)	HT count^a^	HS count^a^	Fold change expression (HT/HS)	*p*-value	Signature (*p* < 0.001)
miR156	bol-miR156a	UGACAGAAGAGAGUGAGCACA	10.64	4.46	2.37	2.16E-16	TRUE
	bol-miR156b	UUGACAGAAGAUAGAGAGCAC	25.38	9.22	2.73	1.97E-46	TRUE
	bol-miR156e	UGACAGAAGAGAGUGAGCAC	10.05	3.89	2.56	1.10E-17	TRUE
	bol-miR156l	UGACAGAAGAGAGUGAGCAC	9.79	3.96	2.45	4.18E-16	TRUE
	bol-miR156n	UGACAGAAGAGAGUGAGCAC	9.05	4.19	2.14	6.61E-12	TRUE
miR157	bol-miR157a	UUGACAGAAGAUAGAGAGCAC	24.09	9.22	2.59	8.77E-41	TRUE
	bol-miR157b	UUGACAGAAGAUAGAGAGCAC	24.01	9.18	2.59	1.02E-40	TRUE
miR166	bol-miR166d*	GGAUUGUUGUCUGGCACGAGG	0.78	1.83	0.42	6.66E-04	TRUE
miR169	bol-miR169f	UGAGCCAAGGAUGACUUGCCG	11.27	5.45	2.05	2.87E-13	TRUE
	bol-miR169m	UGAGCCAAAGAUGACUUGCCG	40.93	13.10	3.10	1.75E-87	TRUE
miR172	bol-miR172d	AGAAUCUUGAUGAUGCUGCAG	22.35	1.37	16.16	1.33E-129	TRUE
miR391	bol-miR391	UUCGCAGGAGAGAUAGCGCCA	0.07	0.65	0.11	3.22E-04	TRUE
miR395	bol-miR395a	CUGAAGUGUUUGGGGGAACUC	2.29	0.11	19.87	4.30E-15	TRUE
	bol-miR395b	CUGAAGUGUUUGGGGGGACUC	39.53	6.78	5.78	3.61E-148	TRUE
	bol-miR395c	CUGAAGUGUUUGGGGGGACUC	37.86	5.75	6.53	9.13E-153	TRUE
miR398	bol-miR398b	UGUGUUCUCAGGUCACCCCUG	1.07	4.27	0.25	1.40E-13	TRUE
	bol-miR398b*	GGGUCGAUAUGAGAACACAUG	5.54	13.64	0.40	1.36E-22	TRUE
miR400	bol-miR400	UAUGAGAGUAUUAUAAGUCAC	0.59	2.63	0.22	1.27E-09	TRUE
miR408	bol-miR408a*	ACAGGGAACAAGCAGAGCAUG	39.86	14.89	2.65	5.64E-69	TRUE
miR827	bol-miR827*	UUUGUUGAUUGACAUCUAUGC	0.07	2.48	0.03	6.05E-18	TRUE

^a^count was normalized as transcripts per million (TPM)

*miRNA* is from the stem–loop precursor miRNAs, which is processed into the miRNA/miRNA* duplex

### Newly identified miRNAs in the libraries of HT and HS genotypes

**Fig. 3:**
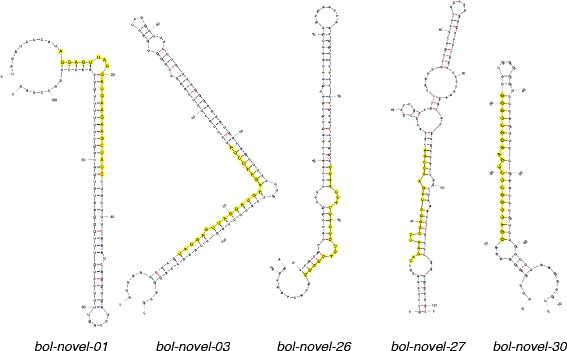
The predicted secondary structures of novel broccoli miRNA precursors. Precursor structures for five novel miRNAs (*bol*-*novel-01*, *bol-novel-03*, *bol- novel-26*, *bol-novel-27*, and *bol-novel-30*) were predicted with yellow indicating the mature miRNA

Novel miRNA candidates were selected on the basis of the secondary structure of precursor sequences, the miRNA/miRNA* duplex and the minimal folding free energy (MFE) value. Using their precursor sequences, their stem-loop hairpin secondary structures were predicted. Most of novel miRNAs are located in the 5′ arm of the hairpin structure. To identify the novel miRNAs associated with heat-tolerant head-forming capacity, the fold-change in expression of novel miRNA candidates were analyzed between the HT and HS genotypes. A total of 24 novel miRNA candidates showed significant differential expression between the HT and HS genotypes (fold change >2 and *P* <0.01) (Table 3). The mean of MFE was–40.31 kcal/mol (range −20.8 to–203.7 kcal/mol) (Additional file 2). Five novel miRNA candidates displayed well-formed secondary structures with lower MFE values were considered potential candidates for further study (Fig. 3 and Additional file 2). Search of corresponding miRNA* sequences showed that 2 of the 24 novel miRNA candidates were expressed in the libraries. The rest of corresponding miRNA* sequences may be hardly found due to their quick degradation or particular spatial/temporal expression pattern. These novel miRNA candidates were inspected in miRNA sequences of other plants for orthologs or homologs, but there was no match in other plant species, which suggests that they are novel and broccoli-specific miRNAs (Table 3). The expression of *novel-16* was 5.5-fold higher in the HS than HT genotype. However, the expression of *novel-01* and *novel-03* was 4.62- and 4.77-fold, respectively, higher in the HT than HS genotype. This is inconsistent with previous observation that novel miRNAs are usually expressed at lower levels, assuming that some novel miRNAs accumulate only in a conditional restricted manner.

**Table 3:** Differentially expressed novel miRNAs in shoot apexes between HT and HS broccoli under heat stress

miRNA	Library miRNA sequence (5'- > 3')	HT count^a^	HS count^a^	Fold change expression (HT/HS)	miRNA*	*p*-value	Signature (*p* < 0.001)
bol-novel-01	AGGAGUUAGGAUGAAGAAGCUAUC	123.42	25.33	4.62	N	0.00E + 00	TRUE
bol-novel-02	AGAGUGAAAAAUAGAGUGAUGAAC	59.73	15.01	3.72	N	3.07E-150	TRUE
bol-novel-03	UAGGAGUUAGGAUGAAGAAGCUA	41.82	8.19	4.77	N	1.29E-131	TRUE
bol-novel-04	AGGGAUAGUUGAGAAUGUGGCUGC	34.54	10.40	3.10	N	1.33E-71	TRUE
bol-novel-05	AAGGAGUGGAGUUUUGAAGAUAGA	48.58	21.25	2.13	N	4.84E-55	TRUE
bol-novel-06	GUGGAAGAAAGAGAAGAUGAUGUG	25.90	8.00	3.02	N	2.35E-52	TRUE
bol-novel-07	GGAGAUAGUUGGGAAUGUGGCUGC	29.66	12.46	2.22	N	4.38E-37	TRUE
bol-novel-09	GGGGCUGUAGAUGCUCUGGACAGC	87.33	213.99	0.38	N	0.00E + 00	TRUE
bol-novel-16	GUUGGCGUUAAGUGAAACGACGUC	9.35	48.49	0.18	N	1.49E-186	TRUE
bol-novel-17	AAGGCACGUGUCGUUGGCUAAG	21.46	68.26	0.29	N	2.83E-170	TRUE
bol-novel-18	GAGACGUUUGAGCUUCGACGC	12.30	45.21	0.25	N	7.55E-132	TRUE
bol-novel-23	GUAUUCUGACGGACAUUCCGACGG	30.44	63.54	0.45	N	6.36E-85	TRUE
bol-novel-25	CACGUGUCGUUGGCUAAGUCC	12.89	36.22	0.33	N	1.50E-78	TRUE
bol-novel-26	UGAAUUACUAUUGCGACGCGG	8.64	27.12	0.30	Y	1.29E-67	TRUE
bol-novel-27	CAGAAUCCGGGCUAGAAGCGA	25.42	50.85	0.47	Y	1.03E-62	TRUE
bol-novel-30	GGAUUGGCUCUGAGGGCUGGG	7.83	21.18	0.35	N	2.73E-44	TRUE
bol-novel-31	CGGAGGUAGGGUCCAGCGGCU	11.41	26.59	0.40	N	2.97E-44	TRUE
bol-novel-33	GAGACAGACUGUACUAGCGACGCU	11.49	25.79	0.42	N	2.50E-40	TRUE
bol-novel-34	AUUGGAUCUGAUUUCUUAAUCGGC	13.89	29.03	0.45	N	8.81E-40	TRUE
bol-novel-35	AGACAGACUGUACUAGCGACGC	12.34	26.74	0.43	N	2.40E-39	TRUE
bol-novel-36	ACUCGUGAUGGCGGACACCUCAGC	10.86	23.16	0.44	N	2.52E-33	TRUE
bol-novel-37	AUUUGGCACUGAUCAAUUGUUCGG	8.24	19.20	0.40	N	2.05E-32	TRUE
bol-novel-39	AUUAUUUGAACUUGGACUCAAUGG	11.86	23.04	0.48	N	1.49E-27	TRUE
bol-novel-40	ACCGUGGUGGUCCAGACUAGAGAC	8.64	16.61	0.49	N	8.45E-20	TRUE

^a^count was normalized as transcripts per million (TPM)

*miRNA* is from the stem–loop precursor miRNAs, which is processed into the miRNA/miRNA* duplex

### Prediction of different expression of miRNA targets in HT and HS

To investigate the biological functions of the known broccoli miRNAs, putative targets of these miRNAs differentially expressed between the HT and HS genotypes were predicted by using psRNATarget [23]. Of the 20 known miRNAs belonging to 10 miRNA families, 97 unique potential targets were identified (Additional file 3). The potential target transcripts were used for a BLASTN search against the Arabidopsis transcript database to identify their homologs in Arabidopsis. A high proportion of miRNA targets belonged to transcription factors, including those encoding squamosa promoter binding protein (SBP, *miR156/157*), nuclear transcription factor Y alpha (NF-YA, *miR169*), the AP2/ERF domain protein (AP2, *miR172*) and the MADS-box transcription factor (MADS, *miR827*) [24]. On analysis of 24 novel miRNAs with differential expression between the two genotypes, 116 potential target genes were predicted (Table 4; Additional file 4). Unlike the conserved miRNAs, the targets of novel broccoli miRNAs were mainly related to transporter activity, kinase activity, and energy pathways. The novel miRNAs may be responsible for regulating diverse biological processes, including developmental processes, signaling transduction pathways and energy metabolic pathways.

**Table 4:** Potential targets of differentially expressed novel miRNAs between HT and HS broccoli under heat stress

miRNA	Predicted targets	Putative function of targets
bol-novel-01	ES999626	Encodes a poly(A) polymerase (PAPS2)
bol-novel-03	ES999626	Encodes a poly(A) polymerase (PAPS2)
bol-novel-06	Bra026115	H^+^-translocating (pyrophosphate-energized) inorganic pyrophosphatase (AVP1)
bol-novel-09	EE566492	Carboxylate clamp-type tetratricopeptide repeat proteins
bol-novel-17	TC211485	Encodes LHCB1.5; Photosystem II type I chlorophyll a/b-binding protein
bol-novel-26	EV080203	Auxin Response Factors 1 (ARF1)
bol-novel-27	TC193973	Jumonji family of transcription factors (JMJ18)
bol-novel-30	Bra005398	Class III HD-Zip transcription factor family (PHB)
bol-novel-31	Bra029290	Encodes OXA1; Integral membrane protein of the thylakoid membrane
bol-novel-34	CD835194	Protein phosphatase 2C
bol-novel-39	EE545548	Hexokinase-like (HKL) proteins
bol-novel-39	TC210139	J domain protein
bol-novel-40	Bra015983	Auxin efflux carrier (PIN1)

### Experimental validation of miRNA-mediated cleavage of their corresponding target mRNAs

**Fig. 4:**
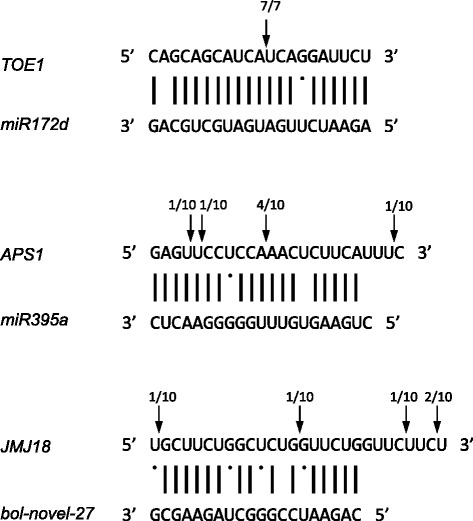
Mapping of target mRNA cleavage sites by RLM 5′-RACE. The cleavage sites are indicated by vertical arrows and the numbers refer to the ratio of the total number of clones sequenced

We performed RLM 5′-RACE experiment to verify the cleavage sites of the miRNA-mediated target genes. As expected, the transcripts of *TOE1* and *APS1* were mostly cleaved in the regions complementary with *miR172d* and *miR395a*. In case of *TOE1* (the targets of *miR172d*), mostly were detected to be cleaved between 11–12 bases relative to the 5′ end of the miRNA. Similar to cleavage of *APS1*, 4 among 10 sequenced clones had a cleavage site between 11–12 nucleotide (Fig. 4). Four cleavage sites of *JMJ18* were located at 1–2, 14–15, 4–5 downstream or 7–8 downstream from the *bol-novel-27* complementary region.

### Validation of the expression levels of broccoli miRNAs

**Fig. 5:**
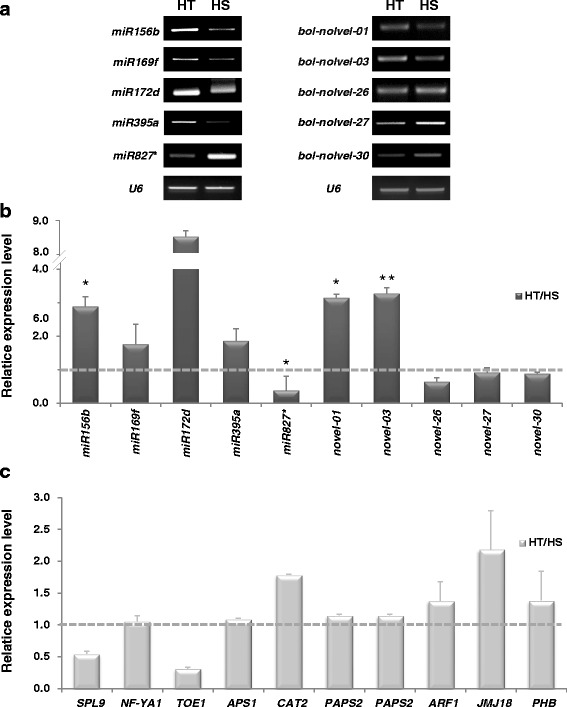
Expression validation of the selected known and novel miRNAs and their putative target genes from shoot apexes of HT and HS broccoli under heat stress. **a** and (**b**) Stem-loop qRT-PCR or RT-PCR showing relative expression of the selected miRNAs. *U6* was used as a loading control (error bars, standard error [SE] of n = 3). **c** qRT-PCR analysis of their putative target genes. *18S* rRNA was used as an internal control. Asterisks denote significant differences from the HT (Student’s *t*-test, *P < 0.05, **P < 0.01)

To confirm the expression profiles of miRNAs obtained from Solexa sequencing, 5 known and 5 novel miRNAs were chosen to analyze their expression using stem-loop semi-quantitative PCR (Fig. 5a) and stem-loop qRT-PCR (Fig. 5b). These miRNAs were selected based on the following reasons: (1) they were high sequencing counts in either HT or HS lines, or (2) they displayed significantly expression differences between HT and HS lines. These potential novel miRNAs were further confirmed by stem-loop RT-PCR (Fig. 5a). The expression pattern of the validated miRNAs by stem-loop qRT-PCR was mostly in agreement with by the sequencing data (Fig. 5b). Overall, these data demonstrated that deep sequencing was a reliable method for detecting and measuring the expression profiles of miRNAs. However, *bol-novel-27*, and *bol-novel-30* displayed different expression patterns by qRT-PCR and sequencing data (Fig. 5b). The discrepancy may be due to (1) the difference in sensitivity of different fluorescent dyes and methods, or (2) the cloning preferences in deep sequencing [19, 25]. Additionally, we confirmed the expression levels of their corresponding targets by qRT-PCR (Fig. 5c). In most of the cases, the inverse expression patterns of miRNAs and their targets have been observed and this is in accordance with the cleavage of target mRNAs mediated by miRNAs. The transcript levels of *SPL9* and *TOE1* were negatively correlated with the accumulation of *miR156b* and *miR172d*, respectively. The abundance of *CAT2* and *ARF1* transcripts increased with the down-regulation of miR827* and *bol-novel-26*, respectively. Contrarily, the expression of *NF-YA1*, *APS1*, and *PAPS* showed a modest increase while the transcript levels of *miR169f*, *miR395a*, *bol-novel-01* and *bol-novel-03* were up-regulated. The transcripts of *JMJ18* and *PHB* were up-regulated, whereas the expression of *bol-novel-27 and bol-novel-30* remained constant.

### Gene ontology of miRNA target genes between the HT and HS lines

**Fig. 6:**
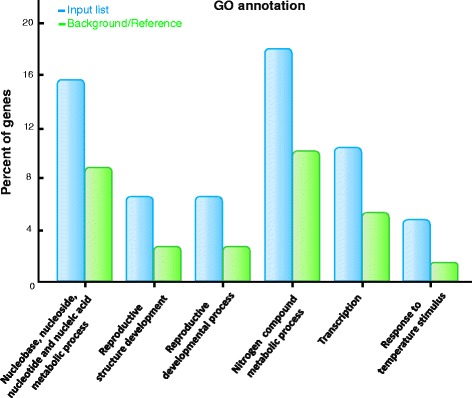
Gene ontology (GO) classification of the predicted target genes of differentially expressed miRNAs between HT and HS broccoli during heat treatment. Percentage of miRNA target genes mapped to the GO term against the input genes. The background/reference list is the percentage of total annotated reference genes mapping to GO terms

These targets from known and novel miRNAs with differential expression between the HT and HS genotypes were further categorized using AgriGO analysis [26]. Significant GO terms were related to post-embryonic development, nitrogen compound metabolic process, reproductive developmental process, response to temperature stimulus, and transcription (false discovery rate [FDR] ≤ 0.05; *p* ≤ 0.001) (Fig. 6; Additional file 5). For biological process, 213 genes were assigned to 37 different categories. The target genes encoding transcription factors such as *AP2*, *TOE1*, *TOE2* and *SPL* were implicated in the reproductive developmental process (Additional files 3 and 5). In addition, the target genes associated with response to temperature stimulus were enriched. Thus, the expression profiles of differentially expressed miRNAs and their targets may contribute to heat-tolerant head-forming capacity in broccoli.

### Expression of floral regulatory and heat shock protein genes between the HT and HS lines

**Fig. 7:**
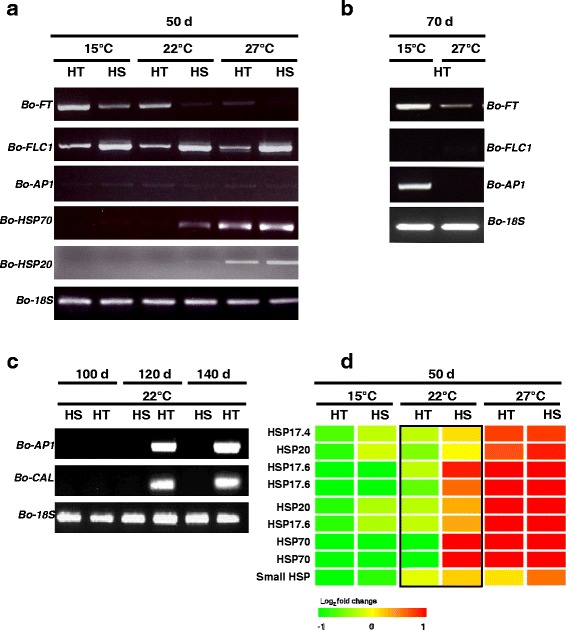
Comparison of floral regulatory and heat shock protein gene expression between the HT and HS lines. **a** RT-PCR analysis of mRNA expression of flowering-time control genes (*Bo-FT*, *Bo-FLC1*, and *Bo-AP1*) and heat stress-responsive genes (*Bo-HSP70* and *Bo-HSP20*) under different temperatures at 50 DPG. *18S* rRNA was used as an internal control. **b** Floral meristem identity genes (*Bo-AP1*) after 70 DPG at 15 °C. **c** Floral meristem identity genes (*Bo-AP1* and *Bo-CAL*) after 100, 120, and 140 DPG at 22 °C. **d** Microarray heatmap of genes encoding plant heat shock proteins, including *HSP70* and *HSP20* gene family. The cDNA sequences encoding proteins were annotated on the basis of *Arabidopsis* genes in TAIR (The *Arabidopsis* Information Resource). The black box indicates the difference in expression level between HT and HS at 22 °C. The log2 expression values derived from 3 biological replications corresponding to each sample were shown

To further address the miRNA-mediated regulation of floral initiation underlying head-forming capacity in the two genotypes at various temperatures, the mRNA expression of the floral regulators *Bo-FT* (positive regulator of flowering), *Bo-FLC1* (negative regulator of flowering), and *Bo-AP1* (floral identity gene) at 50 DPG before head formation were analyzed. At 15 °C, *Bo-FT* was expressed in both HT and HS genotypes. At 22 °C and 27 °C, the expression of *Bo-FT* was predominately expressed in the HT rather than HS genotype. In contrast, the expression of *Bo-FLC1* was higher in the HS than HT genotype at 15 °C, 22 °C and 27 °C. *Bo-AP1* was weakly expressed in both genotypes at all temperatures at 50 DPG (Fig. 7a). The expression level of *Bo-AP1* was induced in the HT genotype at 15 °C at 70 DPG (Fig. 7b). To further explore the temporal expression pattern of floral identity genes in the two genotypes at 22 °C, *Bo-AP1* and *Bo-CAL* expression were detected at different times. At 100 DPG, no *Bo-AP1* or *Bo-CAL* transcripts were detected in either genotype. At 120 and 140 DPG, *Bo-AP1* and *Bo-CAL* transcript levels were higher in the HT than HS genotype (Fig. 7c). From molecular analysis of floral regulators, the HT genotype underwent floral initiation at 50 DPG at 22 °C (Fig. 7a, b and c). Subsequently, the head in HT was developed between 100 and 120 DPG.

We also examined the effects of elevated temperatures on transcript levels of heat stress-responsive genes, *Bo-HSP70* and *Bo-HSP20*, in the two genotypes. The expression level of *Bo-HSP70* was higher in the HS than HT genotype at 22 °C (Fig. 7a). Similarly, the *Bo-HSP20* transcript level was slightly induced in the HS line, but not in the HT genotype at 22 °C (Fig. 7a). Microarray expression profile analysis of *HSP*-related genes also showed higher transcript level of *HSPs* in HS than that of HT at 22 °C (Fig. 7d). Expression level of *Bo-HSP70* and *Bo-HSP20* in HS genotype was 10- and 2-fold higher than those of HT, respectively (Fig. 7d). On the basis of the expression of *HSP* genes, it is suggested that HT was less sensitive to high temperature (22 °C) than HS. Taken together, the head-forming capacity at high temperature of the unique HT line (Fig. 1) was correlated with less sensitivity to heat stress as well as autonomous regulation of *Bo-FT* and *Bo-FLC1* genes (Fig. 7).

### Genotypic variation in response to high temperature during head formation

**Fig. 8:**
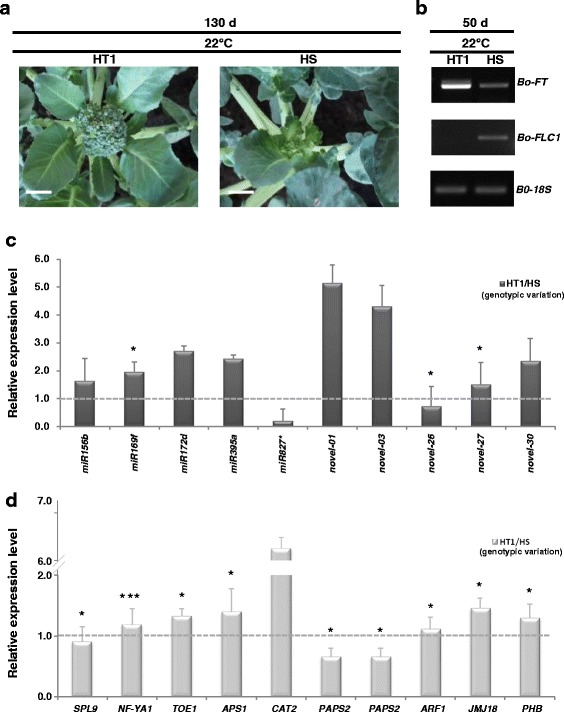
Genotypic variation and characterization of the heat-tolerant 'AVS1' (HT1) and heat-sensitive 'BR1 op' (HS) genotypes in broccoli during head formation under heat stress. **a** Different head-forming capacity of HT1 and HS lines after 128 DPG at 22 °C. Scale bars = 1 cm. **b** Semi-quantitative RT-PCR analysis of mRNA expression of *Bo-FT* and *Bo-FLC1* after 50 DPG at 22 °C. *18S* rRNA was used as an internal control. **c** Stem-loop qRT-PCR showing relative expression of the selected known and novel miRNAs in comparison with HT1 and HS lines. *U6* was used as a loading control. **d** Comparison of the gene expression profiles of their miRNA putative targets between HT1 and HS lines using qRT-PCR analysis. *18S* rRNA was used as an internal control. Asterisks denote significant differences from the HT (Student’s *t*-test, *P < 0.05, ***P < 0.005)

To scrutinize the relationship of genotypic differences and head-forming capacity at high temperature, we obtained the other heat-tolerant ’AVS1′ line (HT1) to test the head-forming capacity at 22 °C at 130 DPG. The HT1 line had a similar phenotype as the HT line with normal head formation under high temperature as compared with the HS line (Fig. 8a). The expression of *Bo-FT* was significantly expressed in the HT1 rather than HS genotype (Fig. 8b). The expression of *Bo-FLC1* was preferentially expressed in the HS than HT1 genotype. Then, we examined the expression of the selected miRNAs and their targets in comparison to HT1 and HS lines using stem-loop qRT-PCR. Eight of these ten miRNAs (miR156b, miR169f, miR172d, miR395a, miR827*, *bol-novel-01, bol-novel-03,* and *bol-novel-26*) shared the same expression patterns in response to high temperature in the HT1 and HT genotypes as compared with the HS genotype (Fig. 8c). The inverse relationship between miRNAs (*miR156b*, miR827*, *bol-novel-01, bol-novel-03* and *bol-novel-26*) and their corresponding targets (*SPL9*, *CAT2*, *PAPS2*, and *ARF1*) expression profiles in the HT1 line was consistent with in the HT line. Changes in *miR169f*, *miR172d, and miR395a* abundance did not have a negative effect on the abundance of *NF-YA1*, *TOE1* and *APS1* (Fig. 8d). It is suggested that enhanced head-forming capacity through the thermotolerance mechanisms could be attributed to genotype-specific expression of miRNAs and their targets.

## Discussion and conclusions

**Fig. 9:**
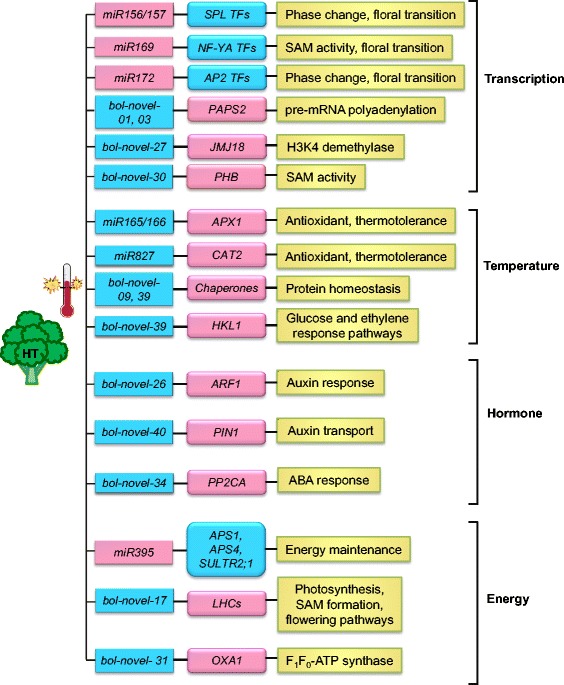
The potential functional network regulated by miRNAs in the HT line of broccoli during head formation in response to heat stress

Several studies have provided strong evidence for a beneficial role of broccoli consumption against cancer [6, 27]. However, global warming and climate changes have reduced broccoli crop yields worldwide [8]. The broccoli inbred lines (HT and HT1) that are capable of producing head at high temperature (in summer without vernalization) described in this study are unique varieties in Taiwan. To understand the molecular mechanisms underlying head formation in broccoli under heat stress, deep sequencing to systematically characterize the miRNAomes in shoot apexes of HT and HS broccoli genotypes were used (Tables 2 and 3). GO classification and functional analysis of predicted miRNA targets resulted in main four subgroups related to transcription, temperature, hormones and energy metabolism (Figs. 6 and 9). Autonomous regulation of the *Bo-FLC1* and *Bo-FT* genes as well as less sensitivity to heat may contribute to floral initiation to produce broccoli heads in the unique HT line as compared to HS (Fig. 7). The targets involved in phase change, SAM activity, reproductive developmental process, cellular redox state, protein homeostasis, hormone signalling pathways and photosynthesis were enriched for heat-tolerant head-forming capacity (Fig. 9).

The targets of the first subgroup of miRNAs are correlated with regulation of SAM development, phase change from vegetative to the reproductive growth, and flowering time [11, 15, 16, 28–31]. With the exception of target of *bol-novel-01 and bol-novel-03*, all of the first subgroup from the predicted target genes is transcription factor. Broccoli *miR156* was predicted to target seven *SPL* transcription factors for the coordination of vegetative development and floral transition in Arabidopsis [15]. The two predicted broccoli *miR169* target genes were homologous to the Arabidopopsis *NF-YA1* and *NF-YA9* transcription factors. The expression of *miR169* was detected in SAM. Overexpression of *NF-YA1* and *NF-YA9* resulted in late flowering [29]. Arabidopsis miR169 family target the AtNF-YA transcription factor to represses *AtFLC*, allowing *AtFT* expression to induce flowering [32]. In this study, it is suggested that accumulation of miR169 in the HT genotype may in turn inhibit the expression of *Bo-FLC1* gene expression (Figs. 5 and 7). The putative targets of *miR172* were four members of the *APETALA2* (*AP2*)-like family of transcription factors, including *AP2*, *TOE1*, *TOE2*, and *SCHLAFMÜTZE* (*SMZ*) [11, 16]*.* The complex pleiotropy of *AP2* functions in maintaining floral meristem size, determining floral organ identity, and affecting flowering time [11, 24]. *TOE1* and *TOE2* are essential for the juvenile-to-adult transition, the vegetative-to-reproductive transition, and the regulation of flowering time, and the *miR172/SMZ* module is a flowering time regulator [11]. Jung et al. (2011) found *35S:156* × *35S:172* and *35S:172* transgenic plants exhibited early flowering, whereas *35S:156* transgenic plants were late flowering as compared with the wild type [28]. In addition, the early flowering phenotype of *35S:172* transgenic plants was temperature-insensitive [24]. It has been reported that downregulation of miR172-regulated AP2-like transcription factors enhanced the expression of FT. FT protein is transported to SAM through phloem and stimulates flowering [33, 34]. In this study, the expression of *miR172* was up-regulated in the HT genotype as compared with the HS (Fig. 5). The expression of *Bo-FT* was predominately expressed in the HT rather than HS genotype at 22 °C (Fig. 7). Therefore, it is suggested that induction of *miR172* in the HT genotype may negatively regulate *AP2*, *TOEs* and *SMZ*, which in turn to induce floral integrator FT. Modulation of *miR172* expression may be a key regulation mechanism linking the thermosensory and flowering-time pathways for heat-tolerant head-forming capacity in broccoli. *bol-novel-01 and bol-novel-03* target transcripts encoding canonical nuclear poly(A) polymerase2, PAPS2, to extend poly(A) tail at the 3′ end of pre-mRNAs [35]. The metabolism of pre-mRNA poly(A) tail is a temperature-dependent process. We suggest that PAPS2 could mediate the formation of pre-mRNA polyadenylation by altered expression of *bol-novel-01* and *bol-novel-03* to protect from heat damage [36, 37]. The target of *bol-novel-27* was predicted to be a jumonji-class transcription factor with H3K4 demethylase activity, *JMJ18*. JMJ18 repressed the expression of *FLC* by reducing the level of H3K4 methylation in *FLC* chromatin to promote the expression of *FT* in companion cells to stimulate flowering [31]. *PHB,* the HD-ZIP III transcription factor gene, was predicted as the target of *bol-novel-30*. Mutation in *PHB* resulted in temperature-sensitive growth defect, altered leaf polarity, and enlarged meristem [30]. Therefore, the first subgroup targets about transcription may trigger a series of developmental processes to regulate meristem development, phase change, and flowering time during head formation under heat stress.

The second subgroup of miRNAs and their potential target genes are related to temperature. Heat stress uncouples enzymes and metabolic pathways which result in the accumulation of excess reactive oxygen species (ROS). Cellular antioxidant defense systems protect plants from heat stress-induced oxidative stress so that they can survive under high temperature [38]. The target genes of *miR165/166* encode the H_2_O_2_-scavenging enzyme, cytosolic ascrobate peroxidase (*APX1*). Overexpression of *cAPX* enhanced tolerance to heat stress in tomato [4]. In addition, the downregulation of *APX1* in the thermosensitivity of the *wrky25* mutant led to increased generation of reactive oxygen species (ROS) under heat stress [39]. Catalase 2 (*CAT2*), a predicted target gene of *miR827* in broccoli, is an ROS detoxifying the enzyme catalase in Arabidopsis [40]. CAT2 is the antioxidant enzyme that removes H_2_O_2_ for heat tolerance and is also connected to light entrainment in the circadian rhythm [41]. Because the circadian clock has a critical role in the flowering transition, the activities of antioxidative enzymes might be necessary for regulating flowering time and thermotolerance mechanisms [42]. Heat stress-induced ROS level can modulate the expression of HSP [43]. In this study, the potential targets of the second subgroup miRNAs are related to antioxidant systems such as CAT2 (Fig. 5). The transcript level of *Bo-HSP70* and *Bo-HSP20* was lower in the HT genotype than that of HS line (Fig. 7). In the HT genotype, it is suggested that the heat stress-induced ROS level could be detoxified by the ROS-scavenging enzymes CAT2 leading to decrease in *HSP* expression. *bol-novel-09* and *bol-novel-39* target transcripts encoding carboxylate clamp-type tetratricopeptide repeat proteins and ER-resident J-protein, respectively. They are involved in ER-resident chaperones and folding enzymes [44–46]. Recent investigations have shown that chaperones are important to facilitate protein homeostasis in response to heat stress [2, 3]. The target of *bol-novel-39* is HEXOKINASE-LIKE1 (*HKL1*), which mediates a cross-talk between glucose and ethylene response pathways [47]. Recent evidence showed the involvement of ethylene (ET) in protecting against heat-induced oxidative stress [48, 49]. Thus, maintaining cellular redox state and protein homeostasis by the ROS/redox signaling network, ethylene response pathways, and chaperones may work in concert to mediate heat-tolerance head formation in broccoli.

In the third subgroup of miRNAs, target genes were associated with homeostasis of hormones, such as auxin and abscisic acid (ABA). Auxin is an important signal for cell division, elongation or differentiation during inflorescence meristem formation [50]. The target mRNA for *bol-novel-26* encodes AUXIN RESPONSE FACTOR 1 (ARF1) in response to auxin to regulate developmental transitions, such as seed germination and flower formation [51]. PIN1, a predicted target gene of *bol-novel-40* in broccoli, was an auxin efflux carrier to maintain auxin gradients for floral meristem formation [50]. ABA pathways potentially contribute to increase thermotolerance in Arabidopsis [49]. The target gene of *bol-novel-34* encodes protein phosphatase 2C (PP2CA), involved in mediating the ABA effects in signaling pathways to coordinate various developmental processes and environmental stresses [52, 53]. The complex regulatory role of miRNAs may participate in auxin and ABA homeostasis, which in turn contributes to head formation and thermotolerance mechanism in broccoli.

The fourth subgroup of miRNAs may act on energy metabolism. The three predicted broccoli *miR395* target genes include two families of genes, ATP sulfurylases (*APS1* and *APS4*) and sulfate transporter 2;1 (*SULTR2;1)*, are involved in sulfate transport and assimilation [54]. Sulfate uptake activity and sulfur assimilation depend on light energy harvesting by consumption of ATP, glutathione, and ferredoxin [55]. The downregulation of *APS1* and *APS4* expression may reduced ATP consumption with maintenance of energy supply in HT under heat stress. Gene encoding the light-harvesting complexes (LHC) was potential targets of *bol-novel-17*. The LHCs of photosystem I (PSI) and PSII are important for photosynthesis to absorb light, transfer energy, and form ATP and NADPH [56]. In tomato, photosynthesis is significantly reduced under heat stress, but the tolerance lines show a normal photosynthetic rate under high temperature [3]. Thus, downregulation of these miRNAs may lead to maintaining energy homeostasis under heat stress. Moreover, loss of function of *PSAD1*, the D-subunit of PSI, resulted in decreased photosynthesis and delayed flowering by blocking the photosynthetic electron flow [57]. The expression of *LHCB1.3/CAB1* through the circadian cycle could also mediate flowering pathways [58]. These evidences imply that these miRNAs may target *LHC* genes, which in turn affect photosynthesis as well as flowering pathways. The target gene of *bol-novel-31* encode an integral membrane protein of the thylakoid membrane, oxidase assembly 1 (OXA1), and are involved in the assembly of the photosynthetic complexes in Arabidopsis [59]. Taken together, these energy-related targets of broccoli miRNAs may play a role in maintaining energy homeostasis for heat-tolerant head formation.

This is the first report of a systematic investigation of miRNAomes between the two genotypes during head formation under high temperature. The expression of miR398b* was greater than that of *miR398b* in the HT library in transcripts per million (TPM). One possible explanation is that the accumulation of miRNA* could regulate genes with sufficient complementarity or regulate its corresponding miRNA precursor itself during specific developmental stages (Table 2). Four cleavage sites of *JMJ18* were located downstream from the *bol-novel-27* complementary region (Fig. 4). The analogous phenomenon was reported in *B. rapa* that the broad variance of cleavage sites of the target mRNAs were observed as the occasional positional heterogeneity for the cleavage site [18]. Additionally, the cleaved products of targets might be very unstable, or secondary siRNAs were triggered by miRNA-mediated cleavage of target mRNAs [12]. Understanding the miRNA-mediated regulation network may help in the molecular breeding of broccoli with heat-tolerant head-forming capacity.

## Methods

### Plant materials and growth conditions

The heat-tolerant 'B295′ (HT) and heat-sensitive 'BR1 op' (HS) broccoli (*B. oleracea* L. var *italica*) were from Kale Biotech. Co. in Taiwan. The other heat-tolerant line 'AVS1′ (HT1) was obtained from Asian Vegetable Research and Development Center in Taiwan. Heat-tolerant 'B295' (HT), 'AVS1' (HT1) and heat-sensitive 'BR1 op' (HS) have nearly isogenic backgrounds [5]. Plants were grown in growth chambers at relative humidity 75 % under an 18-h light/6-h dark photoperiod at 22 °C. Seeds were germinated in vermiculite for 10 days and transplants in 9 cm diameter pot were growth in soil for 50 days. Shoot apexes from 60-day-old plants before head formation were collected and immediately frozen in liquid nitrogen.

### Microarray analysis

Microarray analysis was performed using *Brassica* Gene Expression Microarray (Agilent, Cat. No. G2519F-022520) containing 4× 44 k probe sets on a single chip. Microarray experiments including labeling, hybridizations and data analysis (one sample per chip) were carried out according to the manufacturer’s manual. The trimmed mean target intensity of each array was arbitrarily set to 100. The raw cell intensity data files were imported into Genespring software, which is freely available at Bioinformatics Core for Genomic Medicine and Biotechnology Development at the National Cheng Kung University. The data were normalized with the Robust Multichip Average (RMA) algorithm on the basis of median baseline and converted to log 2 scale to allow the comparison of the three biological replicates performed for each set of experiments. Significantly different gene expression was detected based on the *T*-test implemented in the Genespring software. The Benjamini and Hochberg algorithm calculates false discovery rates (FDR) that are inherently corrected for multiple testing [60]. Genes were considered as being significantly up- and down- regulated if the FDR value for the corresponding probe set was smaller than 0.1.

### sRNA library construction and high-throughput sequencing

sRNA samples were isolated from shoot apexes of HT and HS plants by use of the mirPremier microRNA Isolation Kit (Sigma-Aldrich). sRNA fractions of 18 to 30 nt were purified from 15 % denaturing polyacrylamide TBE-Urea gels with SYBR Safe DNA gel stain (Invitrogen). The size-selected sRNAs were sequentially ligated to the 3′ and 5′ adapters with T4 RNA ligase 2 and T4 RNA ligase, respectively (Illumina Inc.). Reverse transcription was preformed with adapters, and then PCR amplification was performed as described in the Illumina protocol. The PCR products were purified by separation and subsequent elution from 10 % TBE urea polyacrylamide gel. The libraries from the two samples were sequenced by use of Illumina Hiseq2000, which generated paired-end reads of 100 nt (Yourgene Bioscience Co., Taiwan). The sRNA sequence data were deposited in Gene Expression Omnibus (GEO) under accession no. GSE 50546 (http://www.ncbi.nlm.nih.gov/geo/query/acc.cgi?acc=GSE50546).

### Sequence analysis and identification of miRNAs

The raw sequences were removed adaptor sequences, low-quality sequences, low-complexity regions, and shorter than 16 nt or longer than 31 nt. Unique sequences were retained with count numbers of the individual sequence reads. The unique sRNAs were aligned to known non-coding RNAs downloaded from the Rfam database http://www.sanger.ac.uk/science/tools/rfam/ with use of the NCBI BLASTN to discard rRNA, tRNA, snRNA, and snoRNA. To study conserved miRNAs, these remaining sequences were for BLASTN searches against known plant miRNAs from miRBase v 21 [20]. Sequences with identical or < 2 mismatches to *viridiplantae* mature miRNAs were considered potential conserved miRNAs. The remaining sequences were used to search for novel miRNAs in broccoli.

To study novel miRNA candidates, the remaining sRNAs were predicted by using psRobot (http://omicslab.genetics.ac.cn/psRobot/) [61]. *B. rapa* (mustard), BAC-based genome sequence data from GenBank BrGDB154 and the collected *Brassica* expressed sequence tag were as a source genome. The matched sequences including the cluster unions and about 200-bp flanking sequences were defined as potential miRNAs. Those sequences are potentially related to a set of acceptable miRNA precursor sequences and the cluster unions were likely to be novel miRNA families. Their secondary structures were predicted by mfold with miRNA precursor sequences [62]. According to the previously described criteria, novel miRNAs should satisfy the following: (1) a mature sequence localized in one arm of the stem-loop structure and between 19 and 24 nt; (2) the corresponding miRNA* sequence identified; (3) the pre-miRNA sequence folded into an appropriate stem-loop hairpin secondary structure; (4) the MFE of secondary structures ≤ −20 kcal/mol; and (5) no more than 7-nt mismatches in the miRNA:miRNA* duplex [63]. The potential novel miRNA precursors were also aligned with tRNA, rRNA, snRNA or snoRNA and discarded if found similar.

### miRNA target prediction

The potential targets of miRNAs were identified by use of the web-based psRNATarget (the updated version of miRU) (http://plantgrn.noble.org/psRNATarget/) with default parameters [23]. The identified miRNA sequences in broccoli were used as user-submitted sRNAs; *B. napus* DFCI Gene Index and *B. rapa* CDS from the BRAD Brassica Database were used as plant databases. The potential target transcripts were used for a BLASTN search against the Arabidopsis or broccoli transcript database to identify their orthologs/homologs in Arabidopsis or broccoli, respectively [64–66]

### Expression verification of miRNAs by stem-loop qRT-PCR

sRNA samples were isolated from shoot apexes of HT, HT1 and HS plant lines by use of the mirPremier microRNA Isolation Kit (Sigma-Aldrich). miRNAs of 18 to 30 nt were purified from 15 % denaturing polyacrylamide TBE-Urea gels with SYBR Safe DNA gel stain (Invitrogen). Identified broccoli miRNAs were validated and detected using stem-loop quantitative real time-PCR (qRT-PCR) method [67]. Stem-loop primers binds to specific miRNA at the 3′ region owing to the precision conferred by the primer with the exact reverse complement of six nucleotides corresponding to the 3′ end of each miRNA sequence. Quantitative RT-PCR of miRNA includes two steps: a reverse transcription reaction and a real-time quantitative PCR reaction. In the reverse transcription reaction, the miRNA-specific stem-loop RT primer was hybridized to mature miRNA at 65 °C for 5 min and then on ice for 5 min. Each reaction solution contained 0.3 μg of total small RNAs were then added to 3.33 U/μl M-MLV reverse transcriptase, 1× reverse transcription buffer, 2 mM MgCl_2_ and 0.25 mM each of dNTPs (Promega). The total volume of 15 μl of the reaction mix was used to adjust by sterilized RNase-free water. The reverse transcription reactions were conducted in an Eppendorf Mastercycler at 16 °C for 30 min, followed by 60 cycles of 30 °C for 30 s, 42 °C for 30 s, 50 °C for 1 s and were stopped at 85 °C for 10 min. In the real-time quantitative PCR reaction, a miRNA-specific forward primer and a reverse primer were then added to amplify the PCR products. Each reaction contained 1 μl of product from three times diluted reverse transcription reaction, 0.5× SYBR Green, and 500 nM each of miRNA-specific forward and reverse primers in final volume of 20 μl. The real-time quantitative PCR reaction was carried out on a LightCycler480 machine (Roche). The reaction were incubated at 95 °C for 10 min, followed by 35 cycles of 95 °C for 5 s, 60 °C for 10 s and 72 °C for 1 s, using *U6* as the internal control. Two or three biological replicates with two or three technological replicates were performed. The threshold cycle (Ct) was generated by use of the LightCycler software. The sequences of miRNA-specific stem-loop RT primers and miRNA-specific forward primers and a reverse primer are listed in Additional file 6.

### Semi-quantitative reverse transcriptase PCR and quantitative real-time PCR (qRT-PCR)

Total RNA was isolated with use of an RNeasy Plant Mini Kit (Qiagen), and then treated with DNase I (Takara) to remove DNA contamination. About 2 μg of total RNA was reverse transcribed with 1 ml of oligo(dT)15 primers by use of the Transcriptor High Fidelity cDNA Synthesis Kit (Roche). Semi-quantitative reverse transcriptase (RT)-PCR and quantitative real-time PCR (qRT-PCR) were performed using specific pairs of primers (Additional file 6). Semi-quantitative RT-PCR was carried out at 95 °C for 10 min, and then 25–30 cycles of incubation at 94 °C for 10 s, 55–60 °C 30–60 s and 72 °C for 1 min, with a final extension step of incubation at 72 °C for 10 min in an Eppendorf Mastercycler. PCR products were analyzed on a 2 % (w/v) agarose gel. qRT-PCR were performed at 95 °C for 10 min, and then 40 cycles of incubation at 95 °C for 10 s, 55–60 °C 10 s and 72 °C for 6 s in a LightCycler480 machine using SYBR Green PCR master mixture (Roche). A melting curve analysis was applied to check PCR specificity. The comparative threshold cycle (Ct) method was applied for the calculation of fold changes in gene expression. *18S* rRNA was chosen as a reference gene. These experiments were performed two or three biological replicates with two or three technological replicates. The error bars indicate the standard deviation.

### RLM 5′-RACE

The cleavage sites of the miRNA-targeted genes were validated with the RNA ligase-mediated 5′ rapid amplification of cDNA ends (RLM 5′-RACE) assay using the GeneRacer kit (Invitrogen). A modified procedure for 5′-RACE was followed without the 5′ de-capping step. The 5′ RNA adaptor was directly ligated to the purified total RNA. Next, the reverse transcription product was amplified using the 3′ gene-specific reverse primers for each miRNA target gene. Twenty-five cycles of PCR were performed with the above cDNA product as templates, using the 5′ adaptor primer and 3′ gene-specific reverse primer. If necessary, nested PCR amplifications were further amplified with 5′ nested adaptor primer and 3′ nested gene-specific primer. The amplification products were gel purified on a 1 % agarose gel, cloned into a pCR4-TOPO vector (Invitrogen). At least 10 independent clones were sequenced to map the cleavage site. The primers for RLM 5′-RACE assay are listed in Additional file 6.

### Functional assignment of potential targets of differentially expressed miRNAs

The predicted targets of differentially expressed miRNAs were used for a BLASTN search against the known reference database from Arabidopsis. Corresponding reference targets were subjected to AgriGO toolkit analysis for gene ontology categorization [26].

## Availability of supporting data

The datasets supporting the results of this article are included within the article a + nd its additional files.

## Additional files

Additional file 1:
**Information about known miRNAs from deep sequencing small RNAs in broccoli.** (XLSX 64 kb) Additional file 2:
**Sequences of differentially expressed novel miRNAs between HT and HS lines.** (XLSX 28 kb) Additional file 3:
**Target genes of differentially expressed known miRNAs between HT and HS lines.** (XLSX 24 kb) Additional file 4:
**Predicted targets of novel miRNAs with differential expression between HT and HS lines.** (XLSX 32 kb) Additional file 5:
**Gene ontology (GO) classification of the predicted target genes of the differentially expressed known and novel miRNAs (FDR ≤ 0.05;** ***p*** ≤ **0.001).** (XLSX 52 kb) Additional file 6:
**Primers used in** **RT-PCR**, **real-time** **PCR, and 5′-RACE PCR in this study.** (XLSX 17 kb)

## Abbreviations

AP1: APETALA1
CAL: CAULIFLOWER
DPG: Days post germination
FLC: FLOWERING LOCUS C
FT: FLOWERING LOCUS T
GO: Gene ontology
MFE: Minimal folding free energy
NF-YA: Nuclear transcription factor Y alpha
SAM: Shoot apical meristem
SOC1: SUPPRESSOR OF OVEREXPRESSION OF CONSTANS1
SPL: SQUAMOSA PROMOTER BINDING PROTEIN LIKE
TPM: Transcripts per million
